# Evaluation of HIV-1 DNA levels among adolescents living with perinatally acquired HIV-1 in Yaounde, Cameroon: A contribution to paediatric HIV cure research in Sub-Saharan Africa

**DOI:** 10.1016/j.jve.2024.100367

**Published:** 2024-03-30

**Authors:** Aude Christelle Ka'e, Maria Mercedes Santoro, Leonardo Duca, Collins Ambe Chenwi, Ezechiel Ngoufack Jagni Semengue, Alex Durand Nka, Naomi-Karell Etame, Willy Leroi Togna Pabo, Grace Beloumou, Marie Laure Mpouel, Sandrine Djupsa, Desire Takou, Samuel Martin Sosso, Hyppolite K. Tchidjou, Vittorio Colizzi, Gregory-Edie Halle-Ekane, Carlo-Federico Perno, Sharon Lewin, R Brad Jones, Caroline T. Tiemessen, Francesca Ceccherini-Silberstein, Joseph Fokam

**Affiliations:** a Chantal Biya International Reference Centre for Research on HIV/AIDS Prevention and Management (CIRCB), Yaounde, Cameroon; b Department of Experimental Medicine, University of Rome Tor Vergata, Rome, Italy; c HIV Research for Cure Academy, International AIDS Society, Geneva, Switzerland; d University of Antwerp, Antwerp, Belgium; e University of Yaounde I, Yaounde, Cameroon; f Amiens University Hospital, Amiens, France; g Evangelical University of Cameroon, Bandjoun, Cameroon; h University of Buea, Buea, Cameroon; i Bambino Gesu Pediatric Hospital, IRCCS, Rome, Italy; j The Peter Doherty Institute for Infection and Immunity, Melbourne, Victoria, Australia; k Weill Cornell Medicine Graduate School of Medical Sciences, New York, USA; l Centre for HIV and STIs, National Institute for Communicable Diseases, A Division of the National Health Laboratory Service, University of the Witwatersrand, Johannesburg, South Africa; m Central Technical Group, National AIDS Control Committee, Yaoundé, Cameroon; n National HIV Drug Resistance Working Group, Ministry of Public Health, Yaounde, Cameroon

**Keywords:** Adolescents, Cameroon, HIV-1 DNA levels, HIV-1 RNA, CD4 cell count, ART

## Abstract

**Background:**

With the advent of antiretroviral therapy (ART), most children living with HIV in sub-Saharan Africa (SSA) are growing toward adolescence, with scarcity of evidence on the size of viral reservoirs to enhance paediatric cure research strategies. This study aims to compare HIV-1 proviral DNA levels according to virological response among adolescents living with perinatally acquired HIV-1 (ALPHIV) and identify associated-factors in the Cameroonian context.

**Methods:**

In this observational cohort study, HIV-1 RNA viremia and CD4^+^ T-cell count were assessed through RT-PCR and flow cytometry respectively at three time-points over 18 months of observation. At the third time-point, 80 randomly-selected participants were classified as with viremia (≥50 HIV-1 copies/mL; n = 40) or without viremia (<50 HIV-1 copies/mL; n = 40); immune-competent (≥500 CD4^+^ T cells/mm^3^) or immunocompromised (<500 CD4^+^ T cells/mm^3^). Among these participants, total HIV-1 DNA load was quantified through droplet digital PCR using Bio-Rad QX200.

**Results:**

Of the 80 randomly-selected adolescents, median [IQR] age was 15 (13-17) years, 56.2% were female, duration on ART was 9.3 [5.4–12.2] years. Among the 40 viremic ones (median viremia 7312 [283–71482]) HIV-1 copies/ml, 75.0% (30/40) were in virological failure (≥1000 HIV-1 copies/ml), while median of CD4 T cells were 494 [360–793] cell/mm^3^ with 48.8% (39/80) immunocompromised. No significant variation in HIV-1 RNA viremia and CD4 T cell count was observed between the three time-points, and 13.7% (11/80) adolescents remained aviremic and immune-competent throughout (stable adolescents). A positive and moderate correlation (r = 0.59; p < 0.001) was found between HIV-1 DNA levels and HIV- 1 RNA viremia. Regarding the CD4 T cell count, a negative and weak correlation (r = −0.28; p = 0.014) was found with HIV-1 DNA loads only among adolescents with viremia. Starting ART within the first year of life, ART for over 9 years and aviremia appear as predictors of low HIV-1 DNA loads.

**Conclusion:**

Among ALPHIV, high HIV-1 RNA indicates an elevated viral reservoir size, representing a drawback to cure research. Interestingly, early ART initiation and longer ARTduration lead to sustained viral control and limited HIV-1 reservoir size. As limited size of viral reservoir appears consistent with viral control and immune competence, adolescents with sustained viral control (about 14% of this target population) would be candidates for analytical ART interruptions toward establishing paediatric post-treatment controllers in SSA.

## 1 Background

Despite significant efforts in reducing HIV vertical transmission the rate of paediatric HIV-1 infections remains concerning, with about 130,000 [90,000–210,000] new cases reported among children aged between 0 and 14 years old globally in 2022. Sub-Saharan Africa (SSA) bears the heaviest burden, accounting for 109,000 [72,000–169,000] of these new infections within this age group.^1^

While the advent of antiretroviral treatment (ART) has positively impacted the lifespan of most children living with perinatally acquired HIV-1 allowing them to grow into adolescence and adulthood, challenges persist, particularly in low- and middle-income countries (LMICs) with limited treatment options.^2^ These challenges include lifelong treatment exposure, adherence to ART, persistence of immune dysfunction and advent of drug resistance mutations (DRMs).^2^, ^3^, ^4^, ^5^ Moreover, despite the benefits of ART, it does not provide a sterilising HIV-1 cure due to the lifelong persistence of infection within sanctuaries.^6^ Consequently, virological rebound post treatment cessation/interruption remains a concern largely attributed to the existence of reservoir cells, which constitute the main barrier to achieving HIV-1 functional cure or eradication.^6^, ^7^, ^8^

These reservoirs consist of latent but replication-competent integrated HIV-1 DNA within the human genome, capable of persisting in body tissues even under effective ART.^9^ Reservoir sanctuaries are numerous and include gut-associated lymphoid tissue, lymph nodes, liver, genital tract, central nervous system, bone marrow, spleen, lungs, and blood.^10^^,^^11^ To assess reservoirs size, several virological markers are used such as total HIV-1 DNA.^12^

Even though children living with perinatal HIV-1 infection represent a unique population for studying HIV-1 persistence in reservoirs because of the “known timing” of HIV-1 infection and opportunity for early treatment,^13^ adolescents living with perinatally acquired HIV-1 (ALPHIV) also constitute an important population as they are long-term survivors, having reached a partial controller state during periods of non-adherence.^14^ Indeed, adolescence is an important developmental period characterized by puberty during which physical and mental changes observed affect treatment adherence.^15^ It has been shown that difficulties experienced in achieving virological success during adolescence may impact reservoir size and clinical outcomes.^16^ It is worth noting that despite the high burden of paediatric HIV-1 infection in SSA, there still persists a gap in data concerning the quantitative characterization of HIV-1 DNA in reservoirs, especially in the Central African sub-region which is known for its high genetic diversity of HIV-1.^17^^,^^18^ Most of studies reporting data on the quantitative profile of HIV-1 reservoirs on the SSA paediatric populations were conducted in Southern African countries and were focusing on very early treated infants.^13^^,^^19^, ^20^, ^21^, ^22^ These studies have shown that higher CD4^+^ T cell percentage and lower pre-ART HIV-1 viral load are predictors of low levels of HIV-1 DNA^19^; early ART initiation, longer ART duration as well as long-term virologic control reduce levels of HIV-1 DNA.^13^^,^^22^ However, evidence on the dynamics of HIV-1 DNA levels in ALPHIV with late ART initiation is lacking in a geographical sub-region where late HIV-1 diagnosis is still of concern.^23^ To gain insights into the persistence of HIV-1 DNA in sanctuary sites in this vulnerable population, it is essential to know what is the size of the reservoirs (total HIV-1 DNA levels) we are dealing with. Total HIV-1 DNA is a global biomarker of reservoirs that includes integrated and non-integrated viral genomes coding for both competent and defective viruses.^24^^,^^25^ Their levels in the body are clinically relevant providing insights into HIV pathogenesis and predicting disease progression independently of HIV-1 RNA viral load and CD4 T cells count.^26^

With the goal to contribute to HIV-1 cure research strategies in a high HIV-1 diversity setting, this study aimed to compare HIV-1 proviral DNA levels according to ART response among adolescents living with perinatal infection and identify associated factors in Cameroon.

## 2 Material and methods

### 2.1 Study design

An observational cohort-study was conducted among adolescents living with perinatal HIV-1 infection, aged between 10 and 19 years old and enrolled in the EDCTP-Ready study cohort between March 2018 and February 2019 at the Centre-Medical mere et enfant and the Essos Hospital Centre in Yaoundé, Cameroon.

### 2.2 Data and samples collection

Socio-demographic and biological data, including gender, age, age at ART initiation, ART history, ART duration, adherence to treatment and immuno-virological information, were obtained. At three time-points (at baseline, 6 months, and 12 months), 8 mL of peripheral blood were collected in EDTA tubes and sent to the virological laboratory at the Chantal Biya International Reference Centre for Research on HIV/AIDS Prevention and Management (CIRCB). Blood samples were then centrifuged, resulting in isolated plasma and buffy coat, which were subsequently stored at -80 °C pending analysis.

### 2.3 Randomisation of selected samples

**Fig. 1:**
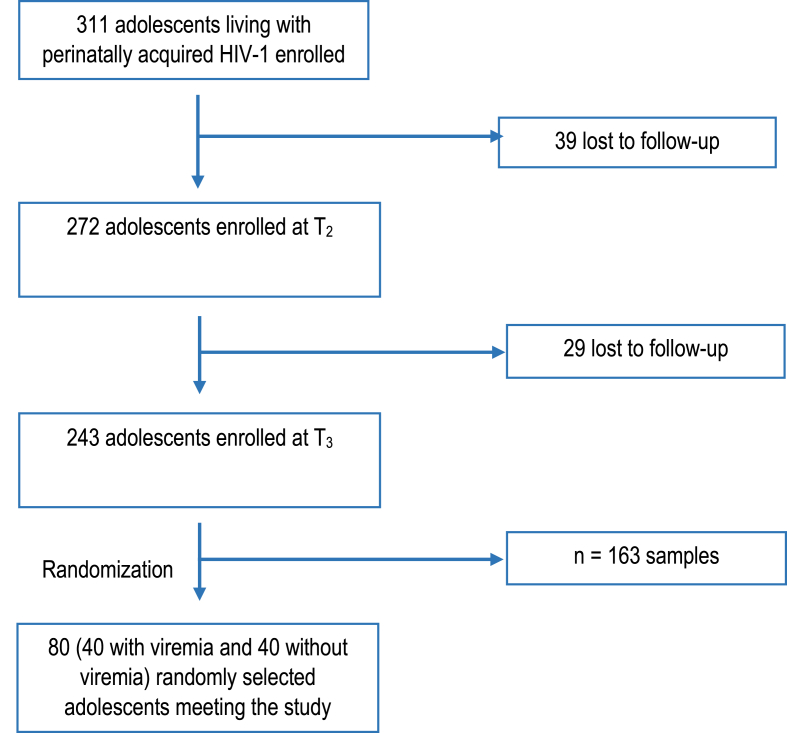
**Flow chart of the sampling selection**.

The EDCTP Ready study started with a cohort of 311 adolescents enrolled according to criteria previously described.^27^ During the second time-point, 39 adolescents were lost to follow-up (LTFU). From the 272 reaching the third time-point, 243 adolescents were enrolled while 29 were LTFU. Among those 243 eligible for the study, a random selection of 80 samples from adolescents with HIV-1 viremia (40) and without viremia (40) were enrolled in the present study (Fig. 1).

A subset of 80 samples (40 with viremia and 40 without viremia) of ALPHIV were randomly selected from the EDCTP Ready study at T_3_ for consideration in the current analysis. T_1_: first time-point; T_2_: second time-point; T_3_: third time-point. LTFU: lost to follow up.

### 2.4 CD4 T cell count

CD4 T cells were measured by flow cytometry with a cytometer, using the CD4 easy count kit as per the manufacturer's instructions (Sysmex Partec CyFlow Counter System GmbH) as previously described.^28^ Participants were then classified based on their CD4 T cell counts into two categories: those considered immunocompetent (CD4 ≥ 500 cells/μL) and those considered immunocompromised (CD4 < 500 cells/μL).

### 2.5 HIV-1 RNA viral load measurement

Circulating HIV-1 viral load of each participant was obtained by RNA extraction and amplification from plasma samples using the Abbott RealTime HIV-1 assay (Abbott Park, IL, USA), which is an *in vitro* reverse transcription-PCR (RT-PCR) assay with a lower and upper detection limit of 40 HIV-1 copies/mL and 10, 000, 000 HIV-1 copies/mL. Analyses were done as per the manufacturer's instructions. Participants were then classified as viremic (≥50 HIV-1 copies/mL) or aviremic (<50 HIV-1 copies/mL).

### 2.6 Quantification of total HIV-1 DNA

Genomic HIV-1 DNA was extracted from 200 μL of buffy coat using the QIAamp DNA Mini Kit (Qiagen, Maryland, USA) according to the manufacturer's instructions. Regarding amplification, each reaction consisted of a 20 mL solution containing 10 mL ddPCR Probe Supermix (Bio-Rad, Hercules, USA), 400 nM primers (human albumin gene and 5-LTR HIV-1 forward and reverse), 125 nM probe, and template DNA pre-diluted at 1/10. The entire 20 μL reaction was loaded into a droplet cartridge (Bio-Rad, Hercules, USA), the cartridge placed into the generator Bio-Rad QX200 and droplets suspended in an emulsion were formed following the manufacturer's instructions as previously described.^29^

The emulsification (40 μL) was transferred into a 96-well reaction plate and sealed as recommended. Total HIV-1 DNA and albumin gene were amplified using the C1000 Thermal Cycler (Bio-Rad, Hercules, USA) following cycling conditions: 10 min hold at 95 °C, 45 cycles of 95 °C for 15 seconds (s) then 60 °C for 60 s.^29^ After amplification, the plate was transferred to a Bio-Rad Droplet Reader from which raw fluorescence amplitude data on cell HIV-1 DNA content (copies/mL) and human albumin gene (copies/mL), genes were extracted from the Quantasoft Software for downstream analysis. Samples were tested in duplicate wells. HIV-1 DNA content in cells (copies/μL) were then normalised into number copies/10^6^white blood cells (WBC) using several parameters such as the level of cell HIV-1 DNA content (copies/μL), factor of dilution, extract volume, and number of WBC (copies/μL of human albumin gene divided by two, assuming that each cell has 2 copies of albumin).

Low level of total HIV-1 DNA was defined as having a value less or equal to the median while high level was defined as having a value above the median.

### 2.7 Statistical analysis

Data was recorded in an Excel spreadsheet and double-checked. Parameters of central tendency (median) and dispersion (interquartile range) were used to describe continuous variables. Categorical variables were described in terms of proportions and frequencies. Chi-square test (or Fisher's test as appropriate) was used to compare proportions, while Mann-Whitney (or Kruskal Wallis as appropriate) test was used to compare median of total HIV-1 DNA levels according to virological and immunological response. Spearman correlation was performed to measure the strength and direction of the association between total HIV-1 DNA, CD4^+^ T cells and HIV-1 RNA viral load. The statistically significant level was set at p ≤ 0.05. Data was analysed using SPSS20.0 and GraphPad Prism 8.

### 2.8 Ethics

The study was carried out in accordance to the Declaration of Helsinki and approved by the National Ethics Committee for Research on Human Subjects (Reference number: 2018/01/981/CE/CNERSH/SP) in Yaounde, Cameroon. Written informed assent from all participants, as well as informed consent from the parents/guardians of children, were obtained.

## 3 Results

### 3.1 Characteristics of the study population

A total of 80 adolescents (95% asymptomatic) were enrolled. The median (IQR) age was 15 (13–17) years; 45/80 (56.2%) of study adolescents were female (Table 1). They had started ART at a median age of 5.5 (3–10) years and were on treatment for a median (IQR) duration of 9.3 (5.4–12.2) years. Regarding their ART history, 45/80 (56.2%) were on first-line (non-nucleoside reverse transcriptase inhibitors-based regimen) and 35 (43.8%) were on second line (protease-based regimen).

**Table 1:** Characteristics of participants at the third study time-point.

Parameters	Categories or medians (IQR)	Overall, N = 80	Participants without viremia, N = 40
*Gender*	Female	45 (56.2%)	23 (57.5%)
	Male	35 (43.8%)	17 (42.5%)
*Age, median (IQR), years*	Median (IQR)	15 (13–17)	15 (13–17)
	Young adolescents	49 (61.2%)	22 (55.0%)
	Old adolescents	31 (38.8%)	18 (45.0%)
*Age at ART initiation (years)*	Median (IQR)	5.5 (3–10)	7 (3–10)
	≤1	6 (7.5%)	3 (7.5%)
	2–5	18 (22.5%)	7 (17.5%)
	>5	24 (30.0%)	17 (42.5%)
	Unknown	32 (40.0%)	13 (32.5%)
*Therapeutic line*	First	45 (56.2%)	20 (50.0%)
	Second	35 (43.8%)	33 (82.5%)
*ART duration (years)*	Median (IQR)	9.3 (5.4–12.2)	8.5 (5.0–12.0%)
	≤9.3	27 (33.8%)	18 (45.0%)
	>9.3	27 (33.8%)	14 (35.0%)
	Unknown	26 (32.5%)	8 (20.0%)
*Adherence to ART*	Yes	54 (67.5%)	33 (82.5%)
	No	26 (32.5%)	7 (17.5%)
*CD4 T cells (cell/mm*^*3*^*)*	Median (IQR)	494 (360–793)	519 (380–848)
	≥500	39 (48.8%)	21 (52.5%)
	350–499	22 (27.5%)	12 (30.0%)
	200–349	14 (17.5%)	6 (15.0%)
	<200	5 (6.2%)	1 (2.5%)
*Plasma HIV-*1 RNA *load (copies/mL)*	Median (IQR)	7312 (283–71482)	–
	<50	40 (50.0%)	–
	50–999	10 (12.5%)	–
	1000–9999	10 (12.5%)	–
	10,000–99999	10 (12.5%)	–
	≥100,000	10 (12.5%)	–
*Drug resistance mutations*	Yes	23 (28.7%)	–
	No	7 (8.8%)	–
	Not applicable	50 (62.5%)	–

Legends. ART: antiretroviral treatment; As concerns therapeutic lines, first line refers to non-nucleoside reverse transcriptase inhibitors-based regimen and second line refers to protease-based regimen. Unknown refers to data that were not recorded in the medical file of the participants. Not applicable in “Drug resistance mutations variable” refer to samples (viral load< HIV-1 1000 copies/mL) which were not eligible for genotyping resistance testing. – refers to “not applicable data information”.

According to virological response at the third time-point of enrolment, for study purpose adolescentspa were stratified as follows: 40 (50.0%) without viremia (viral load< 50 HIV-1 copies/mL) and 40 (50.0%) had viremia (viral load ≥HIV-1 50 copies/mL), with a median viral load of 7312 (283-71 482) HIV-1 copies/ml. Among participants with viremia, a stratification was done as follow: 10/40 (25.0%) with viral load 50-999 HIV-1 copies/mL, 10 (25.0%) with viral load 1000–9999 HIV-1 copies/mL, 10 (25.0%) with viral load 10,000–99,999 HIV-1 copies/mL and 10 (25.0%) with viral load ≥100,000 HIV-1 copies/mL. Therefore, 30/40 (75.0%) were in virological failure (viral load ≥ HIV-1 1000 copies/mL) according to national guidelines, of whom 23/30 (76.67%) harboured DRMs in plasma; further information is reported in Table 1. In terms of HIV-1 subtyping, sequencing was successfully performed for 48 samples, and revealed a broad viral genetic diversity (n = 8 viral clades) of which 5 recombinants: CRF_02AG (72.9%), CRF_A1/G (2.1%), CRF13_cpx (2.1%), A/J (2.1%) and G/F (2.1%) and 3 pure clades: A1, F2 and G (6.2% for each).

The immune status at the third time-point showed a median (IQR) of CD4^+^ T cells of 494 (360–793) cell/mm^3^ and the distribution in the overall population was the same between the group of those who were immunocompetent versus immunocompromised, 41/80 (51.2%) and 39/80 (48.8%), respectively. The aviremic adolescents had a median (IQR) of CD4 T cells was 519 (380–848) cell/mm^3^ and most of them (21 (52.5%) were immune-competent.

### 3.2 Immuno-virological response at the three time-points of enrolment

**Fig. 2:**
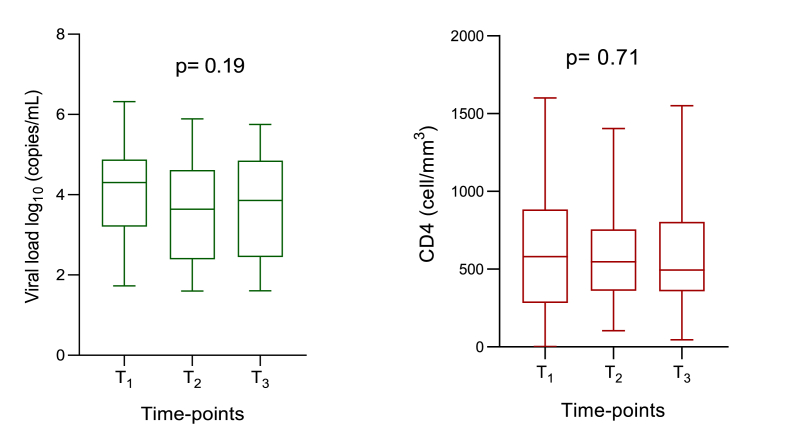
**Trend of HIV-1 RNA load and CD4 T cells at different time-points**. The box plots display the median, IQR as well as the minimum and the maximum of plasma HIV-1 RNA levels (log_10_ copies/mL) and CD4 T cells (cell/mm^3^). The p value reported has been obtained by using the Kruskal-Wallis test. WBC: white blood cells.

In general, these adolescents showed non-significant variations in plasma HIV-1 RNA load (≥50 HIV-1 copies/mL) between the three time-points, with a median [IQR] in log_10_ (copies/mL) of 4.30 [3.20–4.87], 3.64 [2.39–4.61] and 3.86 [2.45–4.85] at time-point 1, 2 and 3, respectively, (p = 0.19; Fig. 2). Nonetheless, a slight increase in number of aviremic adolescents (<50 HIV-1 copies/mL) of 34, 35 and 40, respectively was observed at time-points 1, 2 and 3.

In the same way to that observed for viremia levels, immunological response in the overall population, CD4 T cell counts remained stable, with a median [IQR] cells/mm^3^ of 580 [283–884], 547 [360–756] and 494 [357–803] at time-points 1, 2 and 3 respectively, (p = 0.71; Fig. 2).

### 3.3 Total HIV-1 DNA levels

Regarding the quantitative profile of HIV-1 reservoirs assessed at the third time-point of enrolment, the median (IQR) of total HIV-1 DNA levels were 2.63 (2.26–2.97) log_10_ copies/10^6^ WBC. Respect to gender, there was not significance difference in terms of median [IQR] of HIV-1 DNA levels between males (2.48 [2.24–2.87] log_10_ copies/10^6^ WBC) and females (2.80 [2.26–3.31] log_10_ copies/10^6^ WBC); p = 0.23.

### 3.4 Total HIV-1 DNA and plasmatic HIV-1 RNA levels

**Fig. 3:**
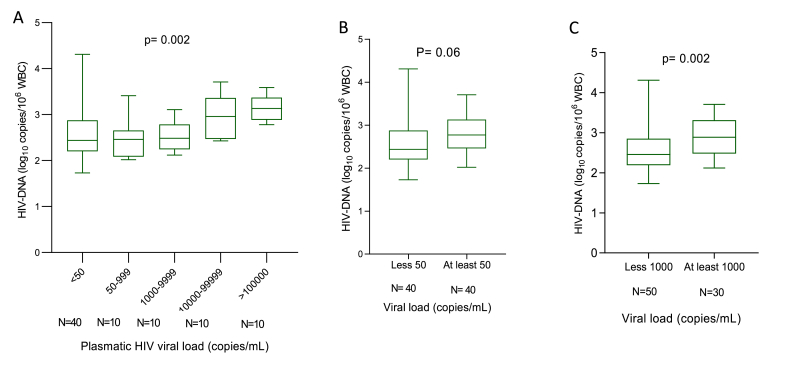
**Trends of the levels of total HIV-1 DNA according to plasmatic HIV-1 RNA load**. The box plots display the median, IQR as well as the minimum and the maximum of total HIV-1 DNA levels (log_10_ copies/10^6^ WBC) categorized according to plasma HIV-1 RNA load (copies/mL). The p value reported have been obtained by using the Kruskal-Wallis test (or Mann-Whitney) test; WBC: white blood cells.

Unsurprisingly, levels of total HIV-1 DNA were significantly increased as HIV-1 RNA load increased (p = 0.002; Fig. 3A). Moreover, considering the virological suppression threshold of 1000 HIV-1 copies/mL as is the case in resource-limited settings like Cameroon, we observed a significantly high levels of total HIV-1 DNA among adolescents with plasma HIV-1 RNA ≥1000 HIV-1 copies/mL as compared to those with less than 1000 HIV-1 copies/mL (Fig. 3C; p = 0.002). Levels of total HIV-1 DNA also appeared to be lower in those under optimal virological control (Fig. 3B), even though it did not reach statistical significance.

### 3.5 Total HIV-1 DNA and CD4 T cells

**Fig. 4:**
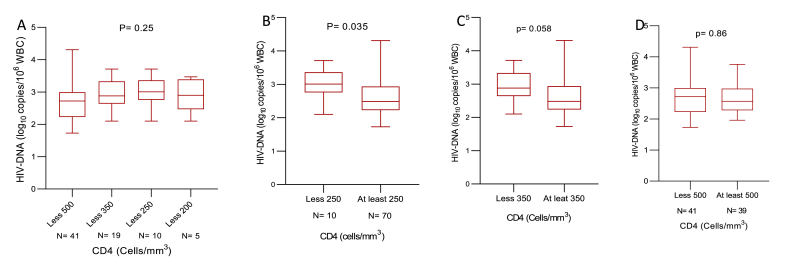
Trends of the levels of total HIV-1 DNA according to CD4 T cells. The box plots display the median, IQR as well as the minimum and the maximum of total HIV-1 DNA levels (log_10_ copies/10^6^ WBC) categorized according to immune status. The p value reported have been obtained by using the Kruskal-Wallis (or Mann-Whitney) test. WBC: white blood cells.

By considering CD4 cell counts, participants were stratified into four categories: <200, 200–350, 351–500 and >500 cells/mm^3^. Even though the levels of total HIV-1 DNA were not significantly different according to ranges mentioned above (Fig. 4A), a decreasing trend of HIV-1 DNA at higher CD4 T cell values in all ranges was found (Fig. 4B–D). Specifically, levels of total HIV-1 DNA appeared to be high among adolescents with immunodeficiency, more precisely in those with CD4 T cells <350 cell/mm^3^ as compared to those having CD4 T cells ≥350 cell/mm^3^ (median [IQR] log_10_ HIV-1 copies/mL: 2.88 [2.64–3.33] vs. 2.48 [2.23–2.94]); (p = 0.05; Fig. 4C).

### 3.6 Correlation between HIV-1 DNA, virological and immunological levels

**Fig. 5:**
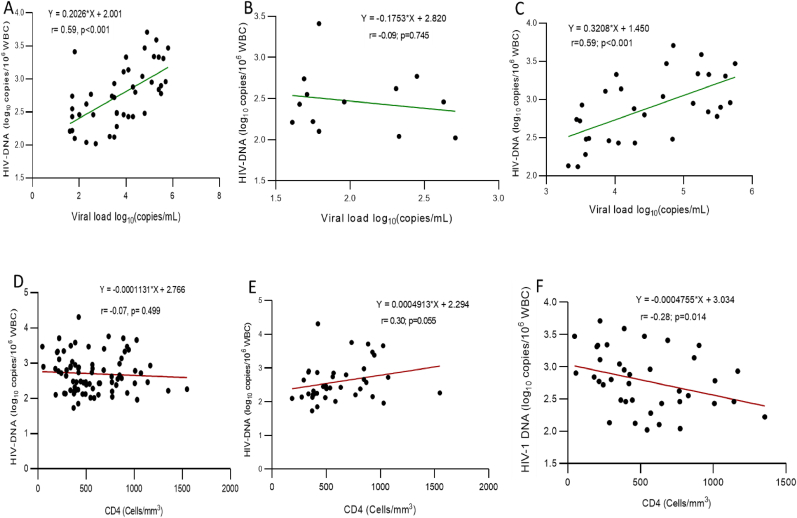
**Correlation between HIV-1 DNA, plasma HIV-1 RNA and CD4 T Cells at time-point three.** WBC: white blood cells. The dots indicate the level of HIV-1 DNA/106 WBC. Spearman correlation was used to evaluate the relationship between total HIV-1 DNA, plasma HIV-1 RNA (in adolescents with detectable viral load; in panel A the correlation was done in the overall population with detectable viral load while in panel B, the correlation was done among those with virological suppression (<1000 copies/mL) vs. virological failure (≥1000 copies/mL) in panel C) and CD4 cells (panel D refers to the correlation for the overall participants while in panel E the correlation was done for participants without viremia vs. with viremia in panel F). The straight line was calculated using linear regression analysis.

A positive and moderate correlation (r = 0.59; p < 0.001) was found between HIV-1 DNA levels and detectable plasma HIV-1 RNA (and more specifically with viral load ≥ HIV-1 1000 copies/mL/) at time-point 3; Fig. 5A. There was no significant correlation between the levels of HIV-1 DNA and HIV-1 RNA in adolescents with virological control (plasma HIV-1 RNA<1000 copies/mL; Fig. 5B).

Regarding CD4 T cell counts, no correlation was observed with HIV-1 DNA levels (Fig. 5) in the overall population. After stratification of the population according to viral load levels, no significant correlation (r = 0.30; p = 0.055) was observed between the CD4 T cell count and HIV- DNA levels in those without viremia (Fig. 5E) while a significant correlation was found among those with viremia (plasma HIV-1 RNA ≥50 copies/mL; Fig. 5F).

### 3.7 Relationship between total HIV-1 DNA levels, socio-demographic and clinical data in the overall population

Adolescents were arbitrarily divided into two groups on either side of the median point of the total HIV-1 DNA levels (2.63 log_10_ HIV-1 DNA copies/10^6^ WBC). Low level of total HIV-1 DNA was defined as having a value less or equal to 2.63 log_10_/10^6^ WBC while high level was defined as having a value above 2.63 log_10_/10^6^ WBC.

In the overall population, participants who started ART before the age of 1 year had low odds of having high levels of total HIV-1 DNA (OR:0.45 [0.35–0.588]; p = 0.026). Additionally, being on ART for a duration of more than 9 years appeared to be associated with low HIV-1 DNA levels (aOR: 0.49 [0.19–1.02]; p = 0.058); Supplementary file 1.

Adolescents with advanced immunodeficiency (CD4 T cells between 200 and 349 cells/mm^3^) at the third time-point of enrolment had a higher odds of high total HIV-1 DNA levels (OR: 4.67 [1.19–18.33]; p = 0.037). While the correlation between total HIV-1 DNA levels and CD4 T cells was not statistically significant in participants with severe immunodeficiency (CD4 T cells < 200), it's worth noting that among the five individuals in this category, four showed elevated levels of total HIV-1 DNA (Supplementary file 1).

Those with virological success (plasma HIV-1 RNA <1000 HIV-1 copies/mL as defined in Cameroon), had very low odds of having high total HIV-1 DNA levels (OR: 0.12 [0.02–0.69]; p = 0.013) whereas those with very high plasma HIV-1 RNA had high odds of high levels of total HIV-1 DNA (OR: 5.20 [2.36–20.60], p = 0.001; Supplementary Table 1).

No significant association was found between total HIV-1 DNA levels and therapeutic regimen as well as treatment adherence. Of note, absence of plasma DRMs was associated with low odds of having high total HIV-1 DNA levels (OR: 0.45 [0.35–0.58]; p = 0.012).

After adjusted odd ratio in multivariate analysis, starting ART before one year of age (independently of virological response) virological control remained significantly associated with low levels of HIV-1 DNA (aOR: 0.53 [0.40–0.73], p = 0.041 and aOR: 0.45 [0.26–0.80], p = 0.035, respectively). Importantly, very high plasma HIV-1 RNA load (>100,000 copies/mL) was associated with high levels of total HIV-1 DNA levels (OR: 3.42 [1.17–16.86]; p = 0.004, Supplementary Table 1).

### 3.8 Relationship between total HIV-1 DNA levels, socio-demographic and clinical data in adolescents without viremia

After performing a subgroup analysis among adolescents without viremia, being on ART for a duration of more than 9 years was a predictor for low HIV-1 DNA levels (OR: 0.13 [0.02–0.77]; p = 0.028). This trend was further confirmed in the multivariate analysis (aOR: 0.41 [0.07–0.63]; p = 0.030, Table 2).

**Table 2:** Total HIV-1 DNA levels according to socio-demographic and clinical data among adolescents without viremia.

Variables	Categories	Total HIV-1 DNA levels (log_10_ copies/10^6^ WBC), N = 40		OR	p value	aOR	p value
		≤2.63 (N = 22)	>2.63 (N = 18)				
*Gender*	Female, n (%)	13 (59.1)	10 (55.6)	1.00	0.538		
	Male n (%)	9 (40.9)	8 (44.4)	1.15 (0.32–4.07)			
*Age (year)*	Median, (IQR)	17 (13–18)	14 (13–16)		**0.042**		
	Young adolescents, n (%)	9 (40.9)	13 (72.2)	1.00			
	Old adolescents, n (%)	13 (59.1)	5 (27.8)	0.26 (0.07–1.01)	**0.048**	1.00	
*Age at ART initiation (year)*	Median, (IQR)	6 (4–8)	8 (3–10)		0.639		
	≤1, n (%)	3 (13.6)	0 (0.0)	1.00			
	2-5, n (%)	3 (13.6)	4 (22.2)	1.81 (0.35–9.40)	0.680		
	>5, n (%)	11 (50.0)	6 (33.3)	0.50 (0.13–1.81)	0.348		
	Unknown, n (%)	5 (22.7)	8 (44.4)	–	–		
*Duration of ART (year)*	Median, (IQR)	10.9 (6.4–12.8)	8.2 (5.3–12.1)		0.387		
	≤9.3, n (%)	8 (40.0)	10 (83.3)	1.00			
	>9.3, n (%)	12 (60.0)	2 (16.7)	0.13 (0.02–0.77)	**0.028**	0.41 (0.07–0.63)	**0.030**
	Unknown, n (%)	2 (9.1)	6 (33.3)	–	–		
*CD4 T cells (Cell/mm*^*3*^*)*	Median, (IQR)	519 (385–771)	422 (281–848)		0.163		
	≥500, n (%)	9 (40.9)	12 (66.7)	1.00			
	350-499, n (%)	10 (45.5)	2 (11.1)	0.15 (0.03–0.82)	**0.035**	0.25 (0.02–2.98)	0.273
	200-349, n (%)	2 (9.1)	4 (22.2)	2.85 (0.46–17.81)	0.381		
	<200, n (%)	1 (4.5)	0 (0.0)	0.53 (0.40–0.72)	1.000		
*Therapeutic lines*	First, n (%)	11 (50.0)	9 (50.0)	1.00			
	Second, n (%)	11 (50.0)	9 (50.0)	1.00 (0.29–3.47)	0.751		
*Adherence*	Good, n (%)	18 (81.8)	15 (83.3)	1.00			
	Poor, n (%)	4 (18.2)	3 (16.7)	0.90 (0.17–4.67)	0.764		

Chi-square (or Fischer) and Mann-Whitney tests were done to evaluate the relationship between qualitative and quantitative variables respectively. Unknown (or not done) categories were not considered when performing test analysis. “Not done” refers to participants among who drug resistance genotyping test was not performed because of plasma HIV-1 RNA levels. Concerning multivariate analysis, only qualitative variables with p value ≤ 0.20 were used to identify parameters independently associated with high levels of total HIV-1 DNA. Young adolescent (10–15 years); old adolescents (16–19 years).

### 3.9 Relationship between total HIV-1 DNA levels, socio-demographic and clinical data in adolescents with viremia

Regarding subgroup analysis among adolescents with viremia, we found that both in univariate and multivariate analyses, those with very high viral loads (≥10 0000 copies/mL) were more likely to have high levels of total HIV-1 DNA as compared to others (aOR: 4.63 [1.27–13.85], p = 0.004). No association was found between total HIV-1 DNA and duration on ART. Even though a slight trend was observed between the CD4 cell count and total HIV-1 DNA, no association was found between these two variables after CD4 T cell stratification. Supplementary Table 2.

### 3.10 Characteristics of adolescents with confirmed HIV-1 control and immunocompetence

Out of the 80 adolescents enrolled, 11 (13.75%, seven males and four females) sustained virological control as well as immunocompetence during the three time-points for 12 months; Table 3. Drug resistance testing was not performed due to their optimal virological control. Without any evidence of hepatitis and/or tuberculosis co-infections these 11 participants could be considered as eligible for analytical antiretroviral treatment interruptions (ATIs). Moreover, out of the 40 adolescents without viremia at the third time-point, no significant difference in the levels of total HIV-1 DNA was observed between those who maintained undetectability and immunocompetence (11 participants described above) throughout the observation period and those with previously fluctuating viral loads (p = 0.709).

**Table 3:** Characteristics of potential eligible study adolescents to the analytic antiretroviral treatment interruption.

Participants ID	PVL-1	PVL-2	PVL-3	CD4 T cells 1	CD4 T cells 2	CD4 T cells 3	HIV-DNA
*CME 011*	ND	ND	ND	817	989	734	3.76
*CME 029*	ND	ND	ND	503	771	1030	1.96
*CME 031*	ND	ND	ND	801	980	1025	3.66
*CME 032*	ND	ND	ND	1047	912	869	2.57
*CME 041*	ND	ND	ND	881	909	848	2.66
*CME 045*	ND	ND	ND	701	992	1550	2.26
*CME 047*	ND	ND	ND	1600	1404	944	3.38
*CME 056*	ND	ND	ND	582	737	814	2.38
*CME 065*	ND	ND	ND	652	625	535	2.41
*CME 066*	ND	ND	ND	930	1208	887	3.71
*CNPS 057*	ND	ND	ND	886	582	1071	2.72

PVL-1 refers to the plasma HIV-1 viral load at the time-point 1 of enrolment; PVL-2 refers to the plasma HIV-1 viral load at time-point 2 of enrolment and PVL-3 refers to the plasma HIV-1 viral load at time-point 3 of enrolment; PVL was expressed in HIV-1 copies/mL. CD4 T cells 1: numeration of CD4 T cells at the first time-point; CD4 T cells 2: numeration of CD4 T cells at the second time-point; CD4 T cells 3: numeration of CD4 T cells at the third time-point; CD4 T cells were expressed in cells/mm^3^. ND: not detectable.

Among the 11 adolescents (all asymptomatic) with maintained HIV-1 viral control and immunocompetence had a total HIV-1 DNA between 1.96 and 3.76 log copies/10^6^WBC (Table 3). In terms of the median total HIV-1 DNA of 2.63 (2.26–2.97) log_10_ copies/10^6^ WBC, most these adolescents have low DNA levels, underscoring their eligibility for a potential functional cure strategy. Moreover, the CD4 T cell count revealed a normal immunity (i.e. CD4 > 500 cells/mm^3^), despite slight fluctuations overtime (Table 3).

## 4 Discussion

With a goal to contribute and address one of the key research goals of the International AIDS Society Global Scientific Strategy 2021 and supporting efforts towards the elimination of AIDS as a pandemic by 2030, the present study provides insights into the quantitative characterization of total HIV-1 DNA in a typical SSA country like Cameroon. Given the framework of ART and the high burden of paediatric HIV-1 infection within this SSA setting,^30^ it represents a unique land of opportunities for setting up baseline investigations that could pave the way for paediatric HIV-1 functional cure research.

The gender distribution was similar among the adolescents enrolled into the study, suggesting an even distribution of the studied parameters. Combined ART was initiated after the first age of the life in most of them (92.5%) suggesting that they were diagnosed in the advanced HIV disease stages, which highlights the ongoing concerns of late HIV-1 diagnosis in SSA and consequently the late initiation of ART.^31^

An analysis of the correlation between HIV-1 DNA levels and plasma HIV-1 RNA load in adolescents with virological failure at the third study time-point (after 18 months of observation) revealed a positive and moderate correlation. Additionally, these were divided into five groups based on plasma HIV-1 RNA load, showing that the levels of total HIV-1 DNA increased as HIV-1 RNA load increased. Similarly, a study conducted in United States have shown a moderate correlation between HIV-1 DNA levels and plasma HIV-1 load in those with viremia^32^, confirming the association between levels of total HIV-1 DNA and viral replication. No correlation was found between plasma HIV-1 RNA and total HIV-1 DNA in adolescents with virological success. By contrast, Hong et al. found a low and positive correlation between total HIV-1 DNA and plasma HIV-1 RNA among adolescents with virological suppression.^32^ These results may suggest the defective nature of most of the persistent proviruses during virological suppression and consequently sub-optimal production of infectious virus as previously described.^25^^,^^33^^,^^34^

Regarding immune status, adolescents were evenly distributed between those with immunocompetent and immunocompromised status, with 51.2% and 48.8%, respectively. Even though some studies have reported association between levels of the HIV-1 DNA and CD4 T cell count,^35^^,^^36^ no significant correlation was found between the levels of HIV-1 DNA and CD4 T cells in the overall population, suggesting that the changes in HIV-1 DNA levels and CD4 T cell counts in response to antiretroviral therapy (ART) do not change in sync or follow a similar pattern.^37^ Moreover, in the absence of viral replication as similarly observed in other paediatric populations on ART, there was still no correlation between these two parameters,^38^ implying that optimal control of HIV-1 replication seemed to affect the association between the HIV-1 DNA levels and CD4 T cell count^37^ inversely to the adult population in whom HIV-1 DNA levels are negatively correlated with the CD4 T cell count, even during non-active viral replication.^39^^,^^40^ This difference observed between the paediatric and adult populations could be explained by the fact that restoration of the CD4 T lymphocyte population depends principally on the generation of naive CD4 T lymphocytes in children while in adults, it drelies mostly on the expansion of the memory CD4 T lymphocyte population.^36^ However, it is worth noting that among adolescents with active viral replication, HIV-1 DNA significantly increased with a decrease of of CD4 T cells, especially in those with <350 cell/mm^3^ as previously reported.^35^^,^^36^

Stratifying adolescents into low and high reservoir size groups in our study based on total HIV-1 DNA levels, we observed that initiating ART before the age of 1 year as well as being on ART for almost 10 years decrease the odds of having high total HIV-1 DNA levels in those without viremia. The decay dynamics of HIV-1 DNA during early ART (within the first 12 weeks of life) in infants has been described previously,^41^, ^42^, ^43^, ^44^ reinforcing the need of very early ART initiation in a context where perinatal transmission of HIV-1 is still of concern.^30^ Additionally, the CHER study has shown that a long duration on ART significantly decrease HIV-1 DNA levels in paediatric populations.^13^

**Fig. 6:**
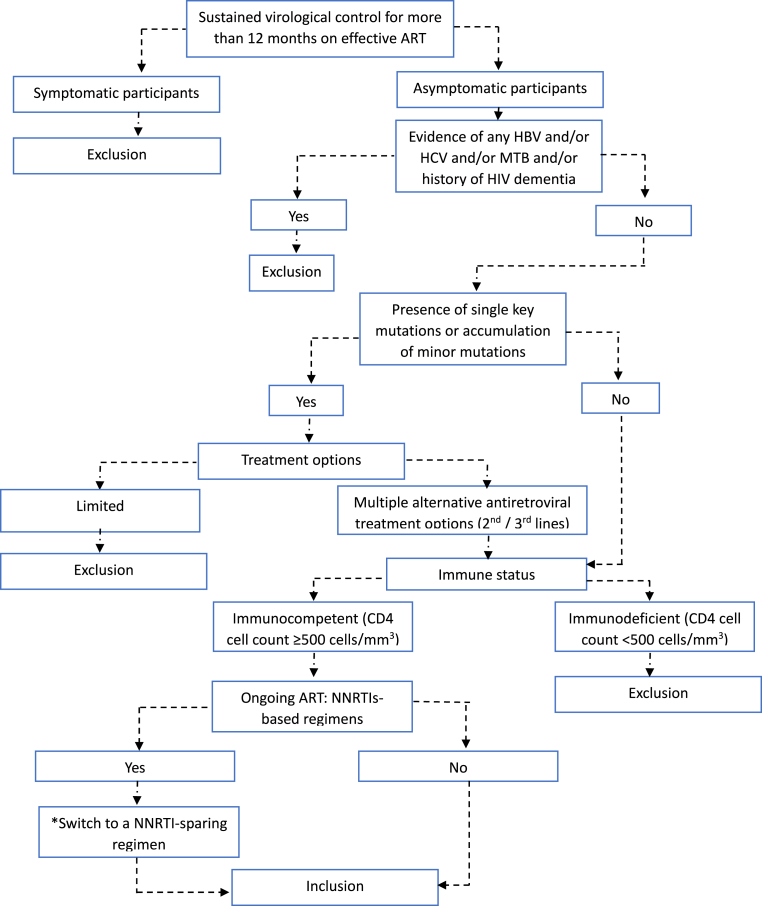
Algorithm for the identification of participants eligible for an analytic treatment interruption (ATI) in Cameroon. HBV: Hepatitis B virus; HCV: Hepatitis C virus; MTB: *Mycobacterium tuberculosis*. *Stopping antiretroviral therapy (ART) including regimens with varying serum half-lives such as non-nucleoside reverse transcriptase inhibitors pose a risk of drug resistance. Thus, participants using these regimens should switch to regimens with short-acting duration (e.g. integrase inhibitors). This algorithm was set using the recommendations for ATIs reported within a consensus meeting between several experts.^14^

When considering challenges of daily ARV intake by adolescents living with HIV, identifying candidates for off-treatment strategy would be relevant. In this study, less than one quarter of them showed a maintained virological control and immune competence without any co-infection across the three time-points. When considering the overall low levels of total HIV-1 DNA among confirmed virologically controlled adolescents with a normal immune status, the consensus recommendations for ATIs,^14^ a tentative evidence-based algorithm for ATI eligibility within the framework of a functional cure strategy among adolescents in the sub-Saharan African context, is summarised in Fig. 6.

Our study limitations include the lack of data such as the CD4 T cell nadir and zenith HIV-1 viral load. Moreover, the lack of total HIV-1 DNA data at the first two time-points did not allow us to further evaluate the dynamics of HIV-1 DNA reservoirs over time within the study population.

## 5 Conclusion

Among ALPHIV receiving ART in Cameroon, high plasma HIV-1 RNA levels predict elevated HIV-1 reservoir size. Most importantly, strategies ensuring very early ART initiation and successful virological response in limiting the reservoir size should be prioritized in this setting with high HIV-1 diversity. Interestingly, sustained virological control, as well as immuno-competence for at least one year, could be main criteria for identifying candidates eligible for an analytical antiretroviral treatment interruption. The above criteria, added to clinical features, may further help to delineate post-treatment controllers towards an HIV functional cure in this SSA setting.

## Funding information

This work was supported by 10.13039/501100001713EDCTP READY Study (TMA2015-CDF1027) implemented at CIRCB and the 10.13039/501100007642Chair of Virology of the University of Rome Tor Vergata, Italy.

## CRediT authorship contribution statement

**Aude Christelle Ka'e:** Writing – review & editing, Writing – original draft, Validation, Formal analysis, Data curation, Conceptualization. **Maria Mercedes Santoro:** Writing – review & editing, Visualization, Validation, Supervision, Conceptualization. **Leonardo Duca:** Writing – review & editing, Validation, Methodology, Investigation, Formal analysis. **Collins Ambe Chenwi:** Writing – review & editing, Visualization, Validation, Investigation, Formal analysis. **Ezechiel Ngoufack Jagni Semengue:** Writing – review & editing, Visualization, Validation, Methodology, Investigation, Formal analysis. **Alex Durand Nka:** Writing – review & editing, Validation, Methodology, Formal analysis. **Naomi-Karell Etame:** Writing – review & editing, Validation, Investigation. **Willy Leroi Togna Pabo:** Writing – review & editing, Validation, Investigation. **Grace Beloumou:** Validation, Investigation, Formal analysis. **Marie Laure Mpouel:** Writing – review & editing, Visualization, Validation, Investigation. **Sandrine Djupsa:** Validation, Methodology, Formal analysis. **Desire Takou:** Writing – review & editing, Visualization, Validation, Investigation. **Samuel Martin Sosso:** Writing – review & editing, Visualization, Validation, Investigation. **Hyppolite K. Tchidjou:** Writing – review & editing, Visualization, Validation. **Vittorio Colizzi:** Writing – review & editing, Visualization, Validation. **Gregory-Edie Halle-Ekane:** Writing – review & editing, Visualization, Validation. **Carlo-Federico Perno:** Writing – review & editing, Visualization, Validation, Methodology. **Sharon Lewin:** Writing – review & editing, Visualization, Validation. **R Brad Jones:** Writing – review & editing, Visualization, Validation, Methodology. **Caroline T. Tiemessen:** Writing – review & editing, Visualization, Validation, Methodology. **Francesca Ceccherini-Silberstein:** Writing – review & editing, Visualization, Validation, Methodology, Funding acquisition. **Joseph Fokam:** Writing – review & editing, Visualization, Validation, Investigation, Funding acquisition, Conceptualization.

## Declaration of competing interest

The authors declared there are no conflicts of interest.

## Data Availability

Data will be made available on request.
