# Supplementary material for: Evaluation of HIV-1 DNA levels among adolescents living with perinatally acquired HIV-1 in Yaounde, Cameroon: A contribution to paediatric HIV cure research in Sub-Saharan Africa
Source: J Virus Erad. 2024 Mar 30;10(1):100367. doi: 10.1016/j.jve.2024.100367 (PMC11004643; doi:10.1016/j.jve.2024.100367)
Supplement: Multimedia component 1 [file mmc1.docx]

Supplementary table 1: Total HIV-1 DNA levels according to socio-demographic and clinical data in the overall population

| **Variables** | **Categories** | **Total HIV-1 DNA levels (log_10_ copies/10^6^ WBC), N=80** | | **OR** | **p value** | **aOR** | **p value** |
| --- | --- | --- | --- | --- | --- | --- | --- |
|  |  | **≤2.63 (N=40)** | **>2.63 (N=40)** |  |  |  |  |
| *Gender* | Female, n (%) | 26 (65.0) | 19 (47.5) | 1 | 0.176 | 1 |  |
|  | Male n (%) | 14 (35.0) | 21 (52.5) | 2.05 (0.83-5.04) |  | 1.95 (0.54-6.99) | 0.302 |
| *Age* | Median, (IQR) | 15 (12-17) | 15 (13-17) |  | 0.638 |  |  |
| *Age at ART initiation (year)* | Median, (IQR) | 4 (2-8) | 7 (3-10) |  | 0.130 |  |  |
|  | ≤1, n (%) | 6 (15.0) | 0 (0.0) | 0.45 (0.35-0.588) | **0.026** | **0.53 (0.40-0.73)** | **0.041** |
|  | 2-5, n (%) | 9 (22.5) | 9 (22.5) | 1.00 (0.382.60)  1.00 (0.38-2.60) | 1.000  1.000 |  |  |
|  | >5, n (%) | 12 (30.0) | 12 (30.0) |  |  |  |  |
|  | Unknown, n (%) | 13 (32.5) | 19 (47.5) | - | - |  |  |
| *Duration of ART (year)* | Median, (IQR) | 10.9 (6.4-12.8) | 8.2 (5.3-12.1) |  | 0.497 |  |  |
|  | ≤9.3, n (%) | 12 (30.0) | 15 (37.5) | 1.40 (0.55-3.55) | 1 |  |  |
|  | >9.3, n (%) | 18 (45.0) | 9 (22.5) | 0.35 (0.14-0.92) | **0.034** | 0.41 (0.19-1.02) | 0.058 |
|  | Unknown, n (%) | 10 (25.0) | 16 (40.0) | - | - |  |  |
| *CD4 T cells (Cell/mm^3^)* | Median, (IQR) | 519 (385-771) | 422 (281-848) |  | 0.163 |  |  |
|  | ≥500, n (%) | 21(52.5) | 18 (45.0) | 1 |  |  |  |
|  | 350-499, n (%) | 15(37.5) | 7 (17.5) | 0.35 (0.12-0.99) | 0.078 | 0.35 (0.36-2.03) | 0.099 |
|  | 200-349, n (%) | 3(7.5) | 11 (27.5) | 4.67 (1.19-18.33) | **0.037** | 3.71 (0.92-10.48) | 0.051 |
|  | <200, n (%) | 1(2.5) | 4 (10.0) | 4.33 (0.46-40.60) | 0.359 |  |  |
| *Viral load (copies/mL)* | Median, (IQR) | 518 (61-4171) | 53361 (5315-231418) |  | **<0.001** |  |  |
|  | <50, n (%) | 23 (57.5) | 17 (42.5) | 1 |  |  |  |
|  | 50-999, n (%) | 8 (20.0) | 2 (5.0) | 0.12 (0.02-0.69) | **0.013** | **0.45 (0.26-0.80)** | **0.035** |
|  | 1 000-9 999, n (%) | 6 (15.0) | 4 (10.0) | 0.43 (0.10-1.83) | 0.295 |  |  |
|  | 10 000-99 999, n (%) | 3 (7.5) | 7 (17.5) | 2.19 (0.48-9.99) | 0.470 |  |  |
|  | ≥100 000, n (%) | 0 (0.0) | 10 (25.0) | 5.20 (2.36-20.60) | **0.001** | **3.42 (1.17-16.86)** | **0.004** |
| *Therapeutic lines* | First, n (%) | 24 (60.0) | 21 (52.5) | 0.73 (0.30-1.78) | 0.652 |  |  |
|  | Second, n (%) | 16 (40.0) | 19 (47.5) | 1.35 (0.56-3.29) |  |  |  |
| *Adherence* | Good, n (%) | 30 (67.5) | 27 (67.5) | 1  1.08 (0.39-2.54) | 0.624 |  |  |
|  | Poor, n (%) | 10 (32.5) | 13 (32.5) |  |  |  |  |
| *Drug resistance* | Yes, n (%) | 9 (22.5) | 14 (35.0) | 1 |  |  |  |
|  | No, n (%) | 0 (0.0) | 7 (17.5) | 0.45 (0.35-0.58) | **0.012** | 0.46 (0.27-0.84) | 0.095 |
|  | Not done, n (%) | 31 (77.5) | 19 (47.5) | - | - |  |  |

Chi-square (or Fischer) and Mann-Whitney tests were done to evaluate the relationship between qualitative and quantitative variables respectively. Unknown (or not done) categories were not considered when performing test analysis. “Not done” refers to participants among who drug resistance genotyping test was not performed because of plasma HIV-1 RNA levels. Concerning multivariate analysis, only qualitative variables with p value≤ 0.20 were used to identify parameters independently associated with high levels of total HIV-1 DNA.
